# Guillain-Barré Syndrome Associated With COVID-19 Infection: A Case Report and Review of Pathophysiology, Risk Factors, and Management

**DOI:** 10.7759/cureus.85414

**Published:** 2025-06-05

**Authors:** Ryan Yang, Muhammad Awan, Shayan Samavati, Alec J Lippmann, Parth Patel, Amit Gupta, Akram Haggag

**Affiliations:** 1 Research, Alabama College of Osteopathic Medicine, Dothan, USA; 2 Medicine, Alabama College of Osteopathic Medicine, Dothan, USA; 3 Clinical Sciences, Alabama College of Osteopathic Medicine, Dothan, USA; 4 Internal Medicine, North Alabama Medical Center, Florence, USA

**Keywords:** albuminocytological dissociation, covid-19, guillain-barrè syndrome, intravenous immunoglobulin (ivig), sars-cov-2 virus

## Abstract

Guillain-Barré syndrome (GBS) is a rare but serious autoimmune neuropathy involving autoimmune destruction of peripheral nerves and rapidly ascending paralysis. Since the start of the COVID-19 pandemic, cases of COVID-19-associated GBS have been increasing in the literature. Although the definitive pathophysiology remains to be studied, SARS-CoV-2 infection is suspected to promote molecular mimicry and induce the development of autoantibodies against peripheral nerves, leading to GBS and its associated manifestations. In this case report, we present a 66-year-old male with a history of coronary artery disease, hypertension, and type II diabetes mellitus, who presented with acute-onset bilateral leg weakness and paresthesia along with a three-day history of fever, chills, and dry cough. The patient tested positive for COVID-19. In addition to his neurological symptoms, cerebrospinal fluid analysis of the patient revealed albuminocytological dissociation, indicating a diagnosis of GBS likely associated with COVID-19 infection. The patient received a five-day course of intravenous immunoglobulin, along with remdesivir and supportive care, for his COVID-19 infection, resulting in significant clinical improvement and complete resolution of his neurological symptoms. This case study examines the potential for GBS to occur in close temporal association with COVID-19 infection and explores the pathophysiology, risk factors, treatment, and preventive measures for COVID-19-associated GBS. Ultimately, our study highlights the importance of clinicians being vigilant for neurological complications, such as Guillain-Barré syndrome (GBS), in patients with SARS-CoV-2 infection, as early diagnosis and intervention can significantly improve patient outcomes and prevent severe complications.

## Introduction

The World Health Organization classified COVID-19, the disease resulting from SARS-CoV-19, as a global pandemic on March 11, 2020, after more than 118,000 cases and nearly 4,300 deaths in 114 countries [1]. While first described as a respiratory illness with symptoms including cough and dyspnea and complications such as pneumonia, it has since become evident that the SARS-CoV-2 virus can cause a range of other systemic conditions, including notable neurological disorders. The neurological complications associated with COVID-19 range from symptoms such as anosmia, ageusia, and lightheadedness to more complicated cases, including stroke, seizures, meningoencephalitis, and necrotizing encephalopathy [2].

Among the impacts of COVID-19 on the nervous system, Guillain-Barré syndrome (GBS) has been identified as one of the most uncommon post-infectious neurological complications. GBS involves the autoimmune destruction of peripheral nerves, the hallmark presentations of which include acute-onset, rapidly progressive bilateral extremity weakness, hyporeflexia, paresthesia, cranial nerve involvement, and cerebrospinal fluid (CSF) albuminocytological dissociation [3]. GBS is classically preceded by an infectious etiology, most commonly of respiratory or gastrointestinal origin. Known triggers of GBS include *Campylobacter jejuni*, Epstein-Barr virus, cytomegalovirus, and Zika virus [4].

Since the outbreak of the COVID-19 pandemic, there has been an increasing number of cases of GBS related to SARS-CoV-2 infection. These cases may present with either a post-infectious onset pattern or an acute-onset pattern. The time interval from the first COVID-19 symptoms to the onset of GBS manifestations is generally 5-21 days [3], suggesting that both post-infectious and para-infectious mechanisms are likely involved. Furthermore, while the incidence of GBS worldwide has remained stable at approximately one to two cases per 100,000 persons per year, systematic reviews have noted a mild increase in GBS incidence associated with COVID-19 infection. For example, a multicenter study in 2020 reported a higher incidence of GBS (2.6 cases per 100,000 per year) after the onset of the COVID-19 pandemic compared to the pre-pandemic incidence of 0.9 cases per 100,000 per year [5].

Although the prevalence of GBS among COVID-19 cases is very low, patients who do present with the disease should be promptly diagnosed and managed due to the risk of fast progression to respiratory failure. There is a need to enhance the awareness and management of GBS. Clinicians should maintain a high level of suspicion for GBS in patients with flaccid paralysis, areflexia, or ascending weakness, especially in patients with concomitant SARS-CoV-2 infection. With early diagnosis of GBS, intravenous immunoglobulin (IVIG) or plasmapheresis can be promptly administered to halt the disease's progression and restore the patient to their baseline neurological function [6].

In this case report, we present a case of GBS occurring in temporal association with COVID-19 infection in a 66-year-old male patient. This case contributes to the still-expanding body of literature that highlights the neuroinvasive potential of SARS-CoV-2 and underscores the importance of recognizing the virus's ability to cause serious autoimmune complications.

## Case presentation

A 66-year-old Caucasian male presented to the emergency department (ED) with complaints of acute-onset bilateral leg pain and leg weakness for one day. The patient had also been experiencing subjective fever, chills, and dry cough for the past three days. He denied recent travel or vaccinations, although he had received the third and final dose of the COVID-19 vaccine about eight months prior. He had a medical history significant for coronary artery disease (CAD), hypertension, type II diabetes mellitus (DM), diverticulosis, and back surgery 23 years ago for a work-related injury involving vertebral disc damage and herniation.

The patient described the leg pain as a burning sensation that radiated from the hips toward the knees and legs bilaterally, causing difficulty in ambulation. The patient also endorsed dyspnea, non-productive cough, fever, and chills. His vital signs on presentation were as follows: blood pressure was 120/69 mmHg, pulse was 79 beats per minute, respiratory rate was 18 breaths per minute, temperature was 98.0°F, and oxygen saturation was 90% on room air. On physical exam, the patient was in mild respiratory distress with diminished breath sounds at the right lung base. His neurological exam was remarkable for paresthesia of the lower extremities bilaterally. The patient denied any dysphonia, dysphagia, or weakness in the upper extremities. Language and comprehension were intact. Pupils were equal and reactive bilaterally. Extraocular motility was intact in all directions. No facial asymmetry was noted. The tongue and uvula were midline. Deep tendon reflexes were 2+ at the right knee and 1+ at the left knee. The patient had 3/5 motor strength in both lower extremities. The decreased motor strength was more apparent distally, and it was not pain-limited, as the patient was still able to move his lower extremities.

The patient tested positive for COVID-19 and negative for influenza A and B. CT imaging of the head in the ED was unremarkable. MRI of the brain initially could not be completed due to the patient’s claustrophobia. His chest X-ray was suggestive of a right lower lobe infiltrate.

Empiric five-day IVIG infusion with diphenhydramine and acetaminophen was initiated due to clinical suspicion of GBS, specifically the acute inflammatory demyelinating polyradiculoneuropathy (AIDP) variant. Gabapentin was additionally started for symptomatic relief. Concurrently, remdesivir was administered for treatment of the COVID-19 infection, intravenous vancomycin for the pulmonary infection, and ipratropium bromide/albuterol sulfate for respiratory distress. The patient continued his home medications except for metformin. Neurology was consulted upon admission, and the patient was scheduled for a lumbar puncture the following day.

On day 2, the patient’s oxygen saturation had improved to 97%, and he described an improvement in his respiratory symptoms. Lumbar puncture was performed for CSF analysis. CSF studies demonstrated high protein levels (81 mg/dL) without an elevation in leukocyte count (1 cell/mm³), further indicating GBS associated with COVID-19 infection. Increased erythrocyte count was also noted on CSF analysis, likely from trauma during the lumbar puncture. Per the cytopathology report, the CSF demonstrated acellular fluid with no pathological findings identified (Table 1).

**Table 1:** Patient’s CSF results and comparison with reference values “H” indicates patient values that are above the reference range. Reference values were obtained from the University of California, San Francisco Health [7]. CSF: cerebrospinal fluid, WBC: white blood cell, RBC: red blood cell, PCR: polymerase chain reaction, IgG: immunoglobulin G, IgM: immunoglobulin M

CSF studies	Reference values	Patient values
Appearance	Clear	Clear
Color	Colorless	Colorless
Leukocyte count (WBC/mm3)	0-5	1
Erythrocyte count (RBC/mm3)	None	33 (H)
Glucose (mg/dL)	50-80	59
Total protein (mg/dL)	15-60	81 (H)
Enterovirus qualitative real-time PCR	Negative	Negative
Herpes I PCR	Negative	Negative
Herpes II PCR	Negative	Negative
JC virus PCR	Negative	Negative
West Nile IgG antibody	Negative	Negative
West Nile IgM antibody	Negative	Negative

**Figure 1:**
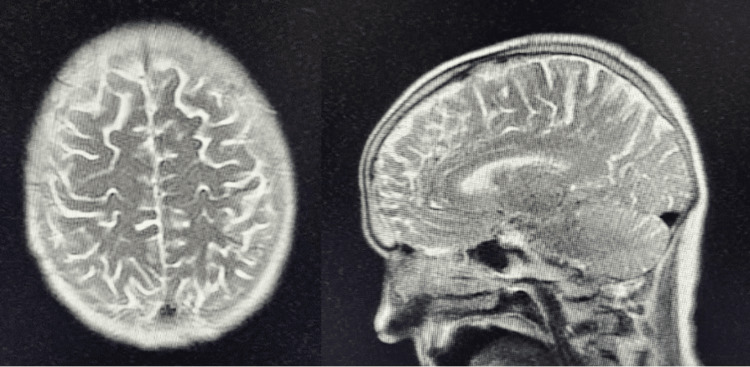
Axial (left) and sagittal (right) T2-weighted imaging of the brain demonstrating scattered nonspecific hyperintensities without evidence of acute abnormalities MRI: magnetic resonance imaging

A second MRI attempt on day 3 of hospitalization was successful, and T2-weighted imaging of the brain revealed scattered nonspecific hyperintensities with no evidence of intracranial hemorrhage, mass, or acute infarct (Figure 1).

**Figure 2:**
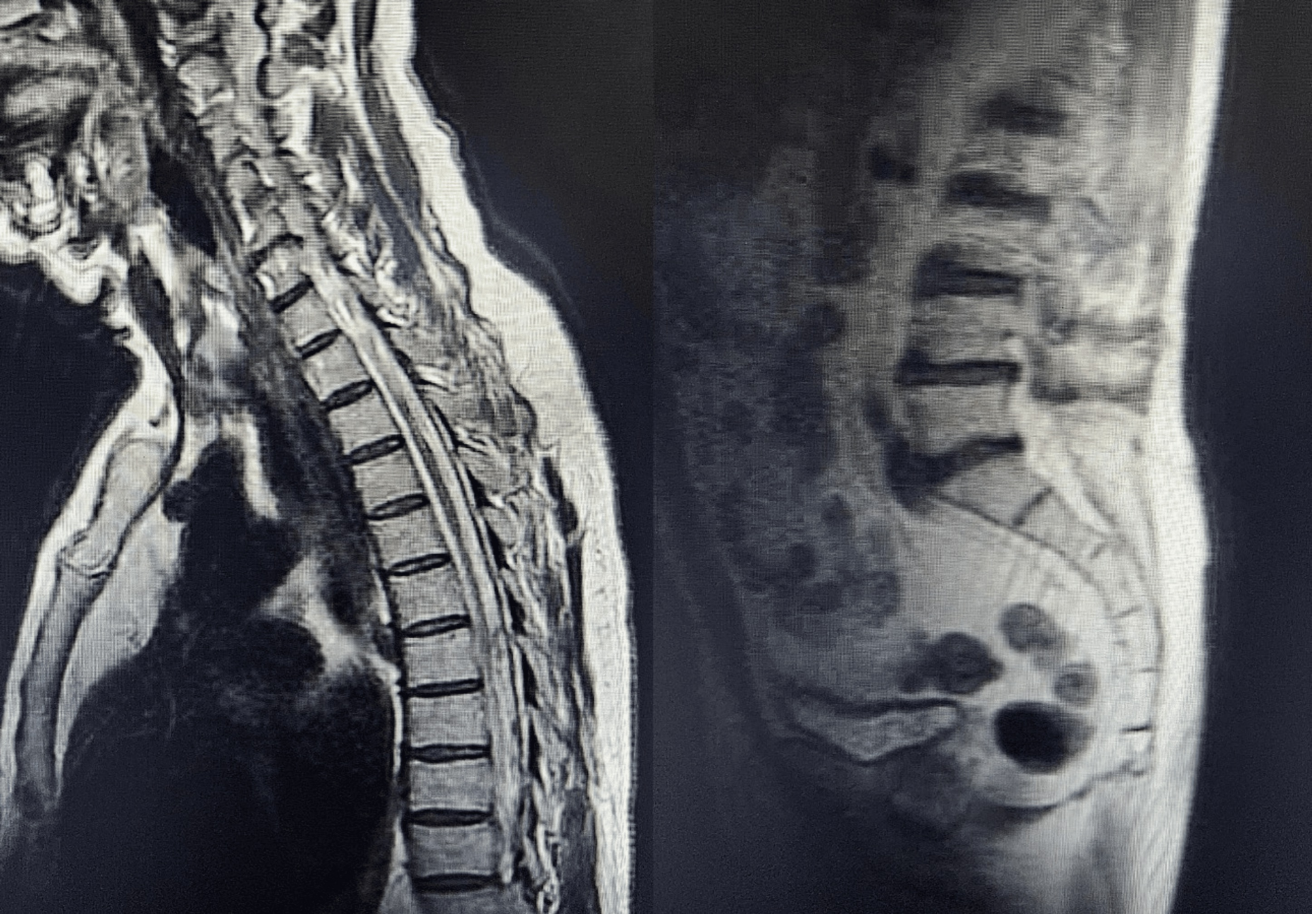
MRI of the thoracic (left) and lumbar (right) spine Thoracic T2-weighted imaging demonstrated a subtle non-enhancing T2-hyperintense mass-like area along the dorsal aspect of the cord extending from T5-6 to the superior endplate of T8 with effacement along the posterior aspect of the cord. Given the lack of enhancement, a mass was considered unlikely. Lumbar spine imaging showed no acute fracture, subluxation, or marrow-replacing process. A linear hyperintensity was seen along the ventral aspect of the distal cord and cauda equina, possibly artifactual. MRI: magnetic resonance imaging

MRI of the thoracic spine was significant for a subtle non-enhancing T2 hyperintense mass-like area along the dorsal aspect of the cord extending from the T5-6 level to the T8 superior endplate level with effacement along the posterior aspect of the cord. Given the lack of enhancement, a mass was considered unlikely. Lumbar spine imaging showed no acute fracture, subluxation, or marrow-replacing process. A linear hyperintensity was seen along the ventral aspect of the distal cord and cauda equina, possibly artifactual (Figure 2).

Blood cultures collected on day 3 showed no growth after 24 hours. The patient reported moderate improvement of his bilateral lower extremity weakness and paresthesia on IVIG treatment, although he complained of occasional tension headaches, dizziness, and blurry vision.

By day 6, the patient’s neurological symptoms had fully resolved, and the physical exam was unremarkable except for a mild dry cough. His vital signs remained stable throughout his hospital course. The patient completed the full course of IVIG and remdesivir on day 7 without complications and had reached maximum clinical benefit from hospitalization. While he was advised to transfer to the rehabilitation center, the patient elected to be discharged home. The patient was informed of the importance of following up with neurology for several months in the case of residual weakness or treatment-related fluctuations, possible manifestations of chronic inflammatory demyelinating polyneuropathy.

## Discussion

This case report details a 66-year-old male who presented with GBS in temporal association with a recent COVID-19 infection. The patient, with a medical history significant for CAD, hypertension, type II diabetes mellitus, diverticulosis, and prior back surgery, developed acute-onset bilateral lower extremity pain and weakness one day after experiencing subjective fever, chills, and a dry cough. The diagnosis of GBS was suspected clinically and confirmed by CSF analysis, which revealed elevated protein levels with a normal cell count. The patient was treated with a five-day course of IVIG and showed significant improvement in his neurological symptoms before being discharged home with recommendations for neurology follow-up. This case contributes to the growing body of literature highlighting the association of SARS-CoV-2 with autoimmune complications such as GBS.

Pathophysiology of COVID-19-associated GBS

COVID-19 is caused by SARS-CoV-2 infection, which is a positive, single-stranded, enveloped ribonucleic acid (RNA) virus. The transmission of infection is primarily through respiratory droplets [8]. SARS-CoV-2 gains entry into host cells and targets the angiotensin-converting enzyme 2 receptor, which is expressed in the respiratory system, gastrointestinal system, and various other tissues [8]. The viral entry initiates a cascade of events that includes the infiltration of proinflammatory markers, such as interleukin-2, interleukin-6, and tumor necrosis factor-alpha, leading to the development of acute respiratory distress syndrome [8].

In addition to the pulmonary complications, COVID-19 has been associated with a neurological phenomenon, GBS, through the mechanism of molecular mimicry. In cases of GBS associated with COVID-19, the most reported variant is the AIDP variant, which is a demyelinating subtype [3]. Regional GBS variants can occur as well. Studies from specific regions have noted a higher rate of facial diplegia [3]. Specifically, research suggests that SARS-CoV-2 infection may lead to the production of anti-ganglioside autoantibodies (i.e., anti-GM1, anti-GQ1b). These autoantibodies may target and destroy peripheral nerves, which are concentrated with gangliosides, ultimately leading to GBS and its manifestations [9,10]. Interestingly, specific anti-ganglioside antibodies appear to correlate with the severity of neurological involvement. For instance, studies indicate that the presence of anti-GM1 antibodies is often associated with more severe GBS cases (more specifically, axonal variants of GBS) [11,12], whereas anti-GQ1b antibodies have been linked to milder clinical presentations and a more favorable long-term prognosis [10]. There are limited studies and evidence on the detection rate of anti-GM1 antibodies before and after COVID-19. Most studies do not suggest a significant change in anti-GM1 antibodies among GBS patients post-COVID-19 compared to their pre-COVID-19 baseline levels. Therefore, more comprehensive serologic studies are needed to determine whether SARS-CoV-2 infection alters the levels of anti-GM1 antibodies in GBS presentations.

Adding another layer of complexity, some instances of COVID-19-associated GBS have presented with atypical antibody profiles compared to those of classic GBS. This raises the possibility of distinct pathogenic mechanisms in these cases. However, the observation that at least one COVID-19-related GBS case exhibited the typical antibody profile seen in GBS unrelated to COVID-19 suggests potential overlap in the underlying immunological pathways [10]. A deeper understanding of these specific antibody profiles in the context of COVID-19-related GBS holds significant implications. Identifying these biomarkers could prove crucial for accurate diagnosis, predicting disease course and potential outcomes, and ultimately informing the development of targeted treatment strategies for individuals experiencing this neurological complication following SARS-CoV-2 infection.

Risk factors for COVID-19-associated GBS

Several studies suggest that certain factors may increase the chances of developing GBS after a COVID-19 infection. One of the key risk factors appears to be age, with people over 50 showing a higher likelihood of being affected [13]. Our patient was 66 years old, which falls into the age group considered to be at higher risk. This finding aligns with a systematic review of 436 post-COVID-19 GBS cases, which reported an average age of approximately 61, reinforcing the association between older age and the development of this condition [2]. A meta-analysis also identified older age as a common risk factor for developing GBS after a SARS-CoV-2 infection [14], suggesting that the changes in the immune system associated with aging may make some individuals more susceptible to this complication. As people age, their immune systems may not respond as effectively to viral infections and can become more susceptible to imbalances, which may increase the risk of autoimmune conditions, such as GBS.

Gender may also play a role; studies have shown that such occurrences are slightly more common in men than in women [13]. Our patient is male, which is consistent with this pattern. Another key factor to consider is the presence of underlying health issues. People with chronic conditions like diabetes or heart disease are more likely to experience serious complications from COVID-19. Our patient had several comorbidities, including CAD, hypertension, and type II diabetes mellitus. A similar case of COVID-19-associated GBS was documented in an older male with chronic myelogenous leukemia, hypertension, and CAD [15], suggesting that GBS often occurs in individuals with underlying health conditions. It is difficult to determine with certainty whether our patient’s existing health problems directly caused GBS after contracting COVID-19. However, having multiple chronic conditions may signal that the body is more vulnerable or reacts more strongly to the infection.

The interval separating the viral prodrome from the neurological deficit is a key clue to causality. In this case, lower-extremity pain and weakness began ≈ 48-72 hours after the first fever and cough, well inside the ≤ 6-week window that large series use to attribute GBS to COVID-19 [16]. Such a window is biologically plausible. During the classic post-infectious pathway, viral epitopes that resemble peripheral-nerve gangliosides stimulate the expansion of class-switched IgG anti-ganglioside antibodies over roughly one to four weeks; these antibodies bind at the nodes of Ranvier, fix complement, recruit macrophages, and produce the segmental demyelination or axonal injury that typifies GBS [6].

When weakness appears within only a few days of the respiratory illness, as in our patient, a para-infectious mechanism is thought to dominate. An early surge of IL-6, IL-1β, and TNF-α transiently disrupts the blood-nerve barrier, while bystander activation of memory B cells (primed by earlier coronavirus exposures) releases low-affinity. These cross-reactive antibodies hasten nerve injury without the usual latent period [17].

Although they differ in tempo, both cascades converge on complement activation and macrophage-mediated myelin stripping. This shared endpoint explains why COVID-19-related GBS can present anywhere along the one- to 42-day spectrum yet retains the pathological hallmarks of “classical” post-infectious GBS.

Diagnosis of COVID-19-associated GBS

We diagnosed GBS in this patient primarily based on his clinical symptoms and CSF analysis. He suddenly developed pain and weakness in both legs, along with tingling and reduced reflexes, a hallmark presentation of GBS. Although muscle weakness and paresthesia of the patient’s lower extremities had not yet fully developed at the time of initial examination, the ascending pattern of weakness in the patient’s legs is a common manifestation amongst patients with GBS. Spotting these clinical signs, especially in patients who recently had an infection, can raise suspicion for the diagnosis of GBS in a timely manner.

Lumbar puncture on hospital day 1 revealed albuminocytological dissociation, with a CSF protein concentration of 81 mg/dL and a leukocyte count of 1 cell/mm³, fulfilling level 2 of the Brighton diagnostic criteria. On this basis, we presumed the AIDP variant, which is the most prevalent electrophysiological subtype in both classic and COVID-19-related GBS, as previously mentioned [3].

We acknowledge that comprehensive electrodiagnostic (EDx) studies, including nerve-conduction velocities and electromyography, represent the reference standard for confirming GBS and distinguishing between demyelinating and axonal variants, distinctions that bear on prognosis and follow-up. Unfortunately, such testing was not obtained: the patient exhibited rapid neurological improvement following initiation of IVIG, and he was discharged to inpatient rehabilitation before laboratory scheduling could be arranged. The absence of electrophysiological data limits definitive subtyping and constitutes the principal limitation of this report. Nevertheless, the concordant clinical picture, classic CSF profile, and close temporal relationship to SARS-CoV-2 infection provide compelling evidence for a diagnosis of COVID-19-associated GBS.

Treatment of COVID-19-associated GBS

Our patient was treated with a five-day course of IVIG, which is one of the first-line treatments for GBS, even in cases tied to COVID-19. Early administration is critical in improving patient outcomes. IVIG works by modulating the immune system, neutralizing the pathogenic antibodies, and potentially inhibiting complement activation, all of which can reduce the autoimmune attack on the peripheral nerves. The patient was also started on remdesivir to treat the COVID-19 infection.

Although remdesivir does not directly treat GBS, managing the underlying viral infection remains an important part of comprehensive care. While most studies show that IVIG tends to lead to positive outcomes, it is worth mentioning that IVIG alone does not always improve survival in patients with GBS. This highlights the need for comprehensive care, especially early in the disease course, since GBS can lead to serious complications like respiratory failure and autonomic dysfunction [18].

Plasmapheresis is another well-established treatment option for GBS. Plasmapheresis functions to filter out harmful antibodies from the plasma. IVIG was used more often than plasmapheresis during the pandemic, likely due to its ease of administration and availability. A systematic review found that out of the patients treated for COVID-19-related GBS, 329 received IVIG, while only 45 were treated with plasma exchange. This difference appears to be driven more by logistical factors than by the effectiveness of the medication, since both treatments are equally effective in accelerating recovery from GBS [19].

Prevention of GBS in the context of COVID-19

Our patient had received three COVID-19 vaccine doses before developing COVID-19 and subsequent GBS. The connection between the vaccine and GBS is not straightforward. It is understood thus far that contracting COVID-19 appears to raise the risk of GBS [3]. However, the risk of GBS from the COVID-19 vaccine depends on the specific type of vaccine. The mRNA vaccines, such as Pfizer-BioNTech and Moderna, have not been linked to a significant rise in GBS cases, and they may even offer some protection by reducing the risk of infection [20]. On the other hand, adenovirus-vector vaccines have been associated with a slightly increased risk of GBS in some studies, particularly after the first dose [20]. A 2024 meta-analysis estimated approximately 0.7 GBS cases per million doses for mRNA COVID-19 vaccines, consistent with the background rate, compared to three to four cases per million for adenovirus-vector vaccines.

Even in the cases where there is a slightly increased risk of developing GBS, the probability is nonetheless very low. A large study demonstrated that contracting COVID-19 was linked to a six times higher risk of GBS, whereas receiving the Pfizer-BioNTech COVID-19 vaccine was associated with a lower risk, with an odds ratio of approximately 0.41 [20]. Therefore, individuals who contracted COVID-19 were far more likely to develop GBS than those who were vaccinated against COVID-19.

Thus, it may be deduced that the benefits of vaccination outweigh the smaller risks. Vaccination against COVID-19 not only assists in the prevention of COVID-19 infection itself but also reduces the risk of complications such as GBS. While there is currently no direct preventive measure against GBS, minimizing exposure to infections known to trigger GBS, such as COVID-19, remains the most effective strategy for prevention. In our patient, staying up to date on vaccines and adhering to public health guidelines may have significantly reduced the risk of further serious complications from GBS and accelerated his recovery.

## Conclusions

Our case report of a 66-year-old male who developed GBS shortly after a confirmed COVID-19 infection highlights the neuroinvasive potential of SARS-CoV-2 and its association with autoimmune complications. Early treatment with IVIG significantly improved his neurological symptoms, underscoring the importance of prompt diagnosis and initiation of appropriate therapy. This case adds to the growing body of evidence linking COVID-19 to GBS. It reinforces the need for clinicians to maintain a high index of suspicion for this neurological syndrome in patients with recent or active COVID-19 infection. Further research into the specific risk factors, antibody profiles, and optimal management strategies for COVID-19-associated GBS is warranted to improve patient outcomes and enhance our understanding of the relationship between a viral pandemic and a serious neurological complication.
